# Concordance of Targeted Sequencing from Circulating Tumor DNA and Paired Tumor Tissue for Early Breast Cancer

**DOI:** 10.3390/cancers15184475

**Published:** 2023-09-08

**Authors:** Chi-Cheng Huang, Yi-Fang Tsai, Chun-Yu Liu, Pei-Ju Lien, Yen-Shu Lin, Ta-Chung Chao, Chin-Jung Feng, Yen-Jen Chen, Jiun-I Lai, Han-Fang Cheng, Bo-Fang Chen, Chih-Yi Hsu, Jen-Hwey Chiu, Ling-Ming Tseng

**Affiliations:** 1 Comprehensive Breast Health Center, Department of Surgery, Taipei Veterans General Hospital, Taipei 11217, Taiwan; chishenh74@gmail.com (C.-C.H.); yftsai@vghtpe.gov.tw (Y.-F.T.); cyliu3@gmail.com (C.-Y.L.); prlain@vghtpe.gov.tw (P.-J.L.); yslin13@vghtpe.gov.tw (Y.-S.L.); tcchao@vghtpe.gov.tw (T.-C.C.); cjfeng@vghtpe.gov.tw (C.-J.F.); s19301084@gmail.com (Y.-J.C.); jilai@vghtpe.gov.tw (J.-I.L.); chfang410512@gmail.com (H.-F.C.); fg2821@gmail.com (B.-F.C.); chiujh10@nycu.edu.tw (J.-H.C.); 2 Division of Breast Surgery, Department of Surgery, Taipei Veterans General Hospital, Taipei 11217, Taiwan; 3 Institute of Epidemiology and Preventive Medicine, College of Public Health, National Taiwan University, Taipei 10617, Taiwan; 4 School of Medicine, College of Medicine, National Yang Ming Chiao Tung University, Taipei 11217, Taiwan; cyhsu@vghtpe.gov.tw; 5 Division of Transfusion Medicine, Department of Medicine, Taipei Veterans General Hospital, Taipei 11217, Taiwan; 6 Division of Chemotherapy, Department of Oncology, Taipei Veterans General Hospital, Taipei 11217, Taiwan; 7 Division of Medical Oncology, Department of Oncology, Taipei Veterans General Hospital, Taipei 11217, Taiwan; 8 Institute of Clinical Medicine, School of Medicine, National Yang Ming Chiao Tung University, Taipei 11217, Taiwan; 9 Department of Pathology and Laboratory Medicine, Taipei Veterans General Hospital, Taipei 11217, Taiwan; 10 Institute of Traditional Medicine, School of Medicine, National Yang Ming Chiao Tung University, Taipei 11217, Taiwan

**Keywords:** targeted sequencing, liquid biopsy, ctDNA, concordance, tumor sequencing, early-stage breast cancer

## Abstract

**Simple Summary:**

The VGH-TAYLOR study comprised a subgroup of early-stage breast cancer patients. Targeted sequencing was performed for both fresh-frozen paraffin-embedded (FFPE) tumor tissue and plasma. Common genes interrogated by both platforms were identified, and the concordance between paired targeted sequencing results from the same individual is reported. Only one-quarter of breast cancers were concordant between tumor and liquid biopsy from the same subject. Early-stage breast cancer might shed less circulating tumor DNA (ctDNA) from the tumor and compromise the detectability of liquid biopsy.

**Abstract:**

In this study, we evaluated the concordance of targeted sequencing between paired ctDNA and matched tumor samples from early breast cancers treated with curative intention. Molecular profiling was performed using the Oncomine Comprehensive Assay v3 and the Oncomine Breast cfDNA Assay v2. The liquid biopsy detection rate was 39% (all-stage breast cancers, n = 612). Among 246 early-stage patients assayed for both ctDNA and matched tumor, the cfDNA assay detected 73 (29.6%) and the comprehensive assay detected 201 (81.7%) breast cancers with at least one alteration (χ^2^ test, *p* = 0.001). In total, 67 (25.6%) cases tested positive on both platforms, while the cfDNA and comprehensive assays detected an additional 10 (4%) and 138 (56%) cases, respectively. The most prevalent mutant genes were *TP53* (68.3%) and *KRAS* (53.5%), while the *PIK3CA* (39.4%), *AKT1* (45.9%), and *ERBB2* (17.1%) mutations constituted biomarkers for FDA-approved therapeutics. Our study showed that tumor tissue should be the source of actionable mutation detection for early breast cancers, considering that the concordance rate between tumor and liquid biopsy was only one-quarter.

## 1. Introduction

Circulating tumor DNA (ctDNA), a component of cell-free DNA (cfDNA), refers to DNA fragments shed from primary tumors in the blood. The phenomenon of ctDNA is not totally understood. Current research shows that it may result from apoptosis, necrosis, or active secretion of tumor cells [1,2]. Once detected, ctDNA can be sequenced and genetic variants revealed. As ctDNA may reflect the entire tumor genome immediately, it has gradually gained attention in recent years for potential clinical application. For example, analysis of ctDNA may be a good tool for the early detection of molecular residual disease, assessment of treatment response, and monitoring of disease progression, thus potentially improving cancer patients’ outcomes [3,4,5]. In addition, ctDNA may also be an effective biomarker as a non-invasive surrogate of tumor burden, further widening its clinical applications, including personalized therapy [6].

Liquid biopsy refers to obtaining plasma samples by drawing blood. Samples can be taken and examined at different time points to monitor changes in tumors during treatment. Analysis of ctDNA obtained through liquid biopsy may greatly change the detection, treatment, and monitoring of cancers [7]. For patients with solid tumors (such as patients with advanced breast cancer), because it is often impossible to obtain metastatic samples by direct biopsy or surgical excision, liquid biopsy is particularly attractive due to its non-invasive nature. However, the clinical application of liquid biopsy for early-stage breast cancer has rarely been addressed. With favorable outcomes and limited disease burden, it is unclear whether ctDNA from liquid biopsy deserves comparable attention regarding the prognosis of breast cancers treated with curative intension.

In this study, we evaluated the concordance of targeted sequencing between ctDNA and paired tumor tissue from early-stage breast cancer patients scheduled for surgery with or without adjuvant therapy. Most studies on liquid biopsy have been conducted for advanced disease, whereas we focused on targeted sequencing for early breast cancer. If high concordance was observed, pre-operative liquid biopsy could serve as a non-invasive surrogate for variants supposed to be revealed in the tumor after definitive surgery. On the other hand, poor concordance might limit the clinical application of liquid biopsy for early-stage breast cancer.

## 2. Materials and Methods

This study evaluated the concordance of targeted sequencing between paired ctDNA and tumor samples from a cohort of early breast cancer patients scheduled for curative therapy in Taiwan using a next-generation sequencing (NGS) assay. 

### 2.1. Study Population

The VGH-TAYLOR study: Comprehensive precision medicine research on the heterogeneity of Taiwanese breast cancer patients, consisting of three years of enrollment and approximately four years of follow-up, has been published elsewhere [8]. Breast cancer patients were assigned into Group 1A: planned to receive surgery as the first-line treatment and followed by adjuvant therapy; Group 2: planned to receive neoadjuvant therapy as the first-line treatment and followed by surgery; and Group 3: diagnosed with de novo and treatment-naive stage IV breast cancer, or stage IV breast cancer with recurrence beyond three years after surgery. In the current study, we focused on a subpopulation of early-stage breast cancer patients with upfront surgery (Group 1A) only.

### 2.2. Targeted Sequencing Panel

**Figure 1:**
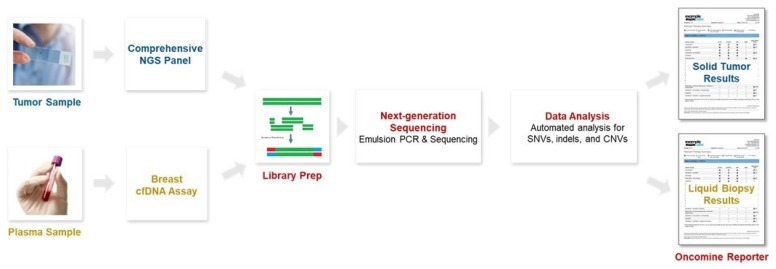
Molecular profiling was performed and potential biomarkers determined using a comprehensive next-generation sequencing (NGS) panel from fresh-frozen paraffin-embedded (FFPE) tissues and a breast-cell-free DNA (cfDNA) assay from plasma.

Molecular profiling was performed and potential biomarkers determined using the Oncomine Comprehensive Assay v3 from fresh-frozen paraffin-embedded (FFPE) tissues and the Oncomine Breast cfDNA Assay v2 from plasma as the form of liquid biopsy. Tumor-only sequencing results of the VGH-TAYLOR study using the comprehensive assay have also been reported [9,10]. The Oncomine Comprehensive Assay is a targeted sequencing panel using FFPE samples, including 161 cancer-relevant genes and types of mutation detected such as frameshift, missense, synonymous, single nucleotide variant (SNV), insertion/deletion (Indel), and copy number variation (CNV). The Oncomine Breast cfDNA Assay detects breast-cancer-derived cfDNA including hotspot genes (~152 hotspots) such as *AKT1*, *EGFR*, *ERBB2*, *ERBB3*, *ESR1*, *FBXW7*, *KRAS*, *PIK3CA*, *SF3B1*, and *TP53*, as well as CNVs of *CCND1*, *ERBB2*, and *FGFR1*. Common genes interrogated by both platforms were identified, and concordance between paired targeted sequencing results from the same subject is reported (Figure 1). Liquid biopsy was carried out at the time of cancer diagnosis, while tumor tissue was obtained from definitive surgery (Group 1A). 

### 2.3. Nucleic Acid Extraction 

Cell-free DNA was obtained from the plasma fraction of a single 10 mL tube of whole blood. The extraction of cfDNA was performed with the QIAamp circulating nucleic acid kit (QIAGEN, Hilden, Germany). The thawed plasma was centrifuged at 16,000× *g* for 10 min at 4 °C. A total of 5 mL supernatant of centrifuged plasma was transferred into a 50 mL tube with 500 µL QIAGENE Proteinase K and 4 mL Buffer ACL without carrier RNA. The mixture was pulse-vortexed for 30 s and then incubated at 60 °C for 30 min. A total of 9 mL Buffer ACB was added into the lysate and mixed well by pulse-vortexing for 15–30 s. The mixture was incubated for 5 min on ice and then applied to the QIAamp Mini column and drawn through the column by a vacuum pump for DNA capture. The captured DNA was washed by 600 µL Buffer ACW1, 750 µL ACW2, and 750 µL ethanol (96–100%) sequentially, and the column was centrifuged at 20,000× *g* for 3 min to remove the remaining wash buffers. The column was incubated at 56 °C for 10 min to dry the membrane completely. Buffer AVE (50 µL) was applied to the dry membrane to elute cfDNA. The eluted DNA was collected in the collection tube by centrifugation at 20,000× *g* for 1 min. The amount of cfDNA harvested was quantified with the Qubit dsDNA HS assay kit (Thermo Fisher Scientific, Waltham, MA, USA). Target enrichment sequencing of cfDNA was carried out following the standard procedures provided by the manufacturer. To achieve 0.1% limit of detection (LOD), 20 ng of input cfDNA was required in this study.

### 2.4. Library Preparation and Variant Calling

Library generation followed the standard protocols: 2–3 libraries (depending on the required read depth) were multiplexed for templating on the Ion OneTouch 2 System and subsequently sequenced on the Ion PGM System using the Ion 318 Chip Kit. Libraries were constructed using the Oncomine Breast cfDNA Assay v2 and the Oncomine Comprehensive Assay v3. Variant detection was performed by the Torrent Variant Caller plugin (version 5.10.0.18)in the Torrent Suite Software (version 5.10.0) or Ion Reporter Software (version 5.6). Additional annotations for actionability and OncoPrinter visualization were carried out using the OncoKB database and ESMO Scale for Clinical Actionability of molecular Targets (ESCAT) criteria [11,12,13].

## 3. Results

The primary outcomes were actionable mutations determined by the genetic profiling of Taiwanese breast cancers.

### 3.1. Detection Rate of Liquid Biopsy (Full Cohort)

**Figure 2:**
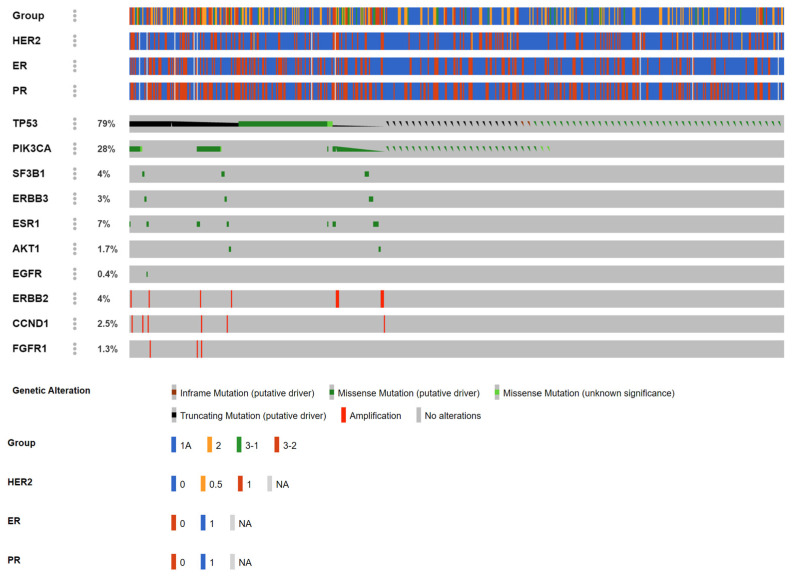
Mutational landscape of the whole VGH-TAYLOR cohort (n = 612) with the Oncomine Breast-cell-free DNA (cfDNA) assay. Group 1A: surgery followed by adjuvant therapy; Group 2: neoadjuvant therapy followed by surgery; Group 3-1: de novo stage IV; Group 3-2: late recurrence (beyond 3 years) after curative surgery (HER2: human epidermal growth factor receptor II, ER: estrogen receptor, PR: progesterone receptor).

We report the mutational landscape of 614 liquid biopsy samples (biopsy times: 1–4) from 494 patients (full VGH-TAYLOR cohort) interrogated with the Oncomine Breast cfDNA assay, which has not been reported previously; 239 out of 614 samples had at least one mutation (39%, Figure 2). *TP53* constitutes the most common variant (79%), followed by *PIK3CA* (28%). Other mutations took place in less than 5% of the study population. Mutual exclusivity was also observed between *TP53* and *PIK3CA* (Log2 Odds Ratio: −2.756, *p*-value and q-value < 0.001).

The distributions of altered genes, as well as amino acid change, functionality, and relevant clinical features are summarized in Table 1. It deserves notice that all *AKT1* p.E17K mutations came from estrogen receptor (ER)-positive/human epidermal growth factor receptor II (HER2)-negative breast cancers, and most were diagnosed with late-stage disease (Groups 3-1 and 3-2, n = 7). All *CCND1*-amplified cases were clinically ER+ and were late-stage (Group 3-2, n = 9), and all *EGFR* p.L861Q samples were late-stage (Group 3-2, n = 7) as well as *FGFR1* amplifications (Group 3-2, n = 7). All *ERBB2*-amplified breast cancers were HER2-positive (over-expression) clinically, with most being ER- and progesterone receptor (PR)-negative and were diagnosed evenly with early- (Group 1 and Group 2A, n = 9) and late-stage disease (n = 7). On the other hand, most *ERBB3* mutations occurred in breast cancers without HER2 over-expression. *ESR1* mutations were exclusively detected in late-stage breast cancers (Group 3-1 and Group 3-2, n = 38), and roughly two-thirds were ER/PR-positive. Hotspots for *PIK3CA* mutations included p.E542K, p.E545K, and p.H1047L and were distributed evenly between early and late stages, with almost three-fourths being ER-positive. *SF3B1* p.K700E mutations were prone to occurring in ER+/PR+/HER2- and early-stage breast cancers. Finally, *TP53* was the most common mutant gene in the current study and comprised missense, truncating, and inframe mutations. 

### 3.2. Early-Stage Breast Cancer Cohort: Concordance between Tissue and Liquid Biopsy

At the time of data lock (September 2021), there were 728 breast cancer patients enrolled in the VGH-TAYLOR study who underwent the tissue-based Oncomine Comprehensive Assay, with 767 samples sequenced (1–2 times per subject). Using unique ID, patients with only one liquid biopsy and only one comprehensive assay were selected (Group 1) in an effort to evaluate the concordance between tissue- and liquid-based samples from the same individual, eliminating repeated measures from the cfDNA assay to provide an unbiased comparison. Finally, 246 patients in Group 1 with 1:1 matching of the comprehensive and cfDNA assays were ready for further analyses. The median age was 56 (range: 31–93, SD: 12.2), and the stage distributions were stage I (n = 96, 39%), stage II (n = 121, 49%), and stage III (n = 28, 11%). Regarding immunohistochemistry (IHC) subtypes, there were 179 hormone receptor (HR)-positive/HER2-negative, 23 HR+/HER2+, 32 HR-/HER2-, and 12 HR-/HER2+ breast cancers, and 59 patients had a family history of breast cancers. 

**Figure 3:**
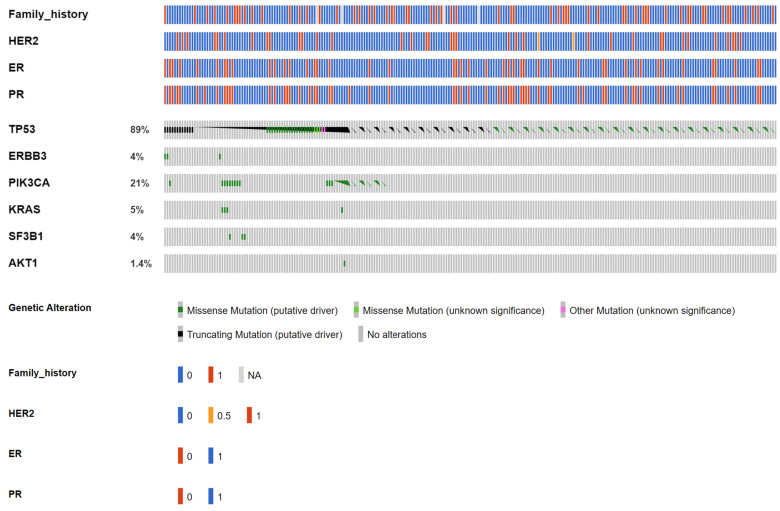
OncoPrinter of circulating tumor DNA (ctDNA) targeted sequencing from 246 early breast cancers. Seventy-three patients reported at least one mutation, which was also the denominator when calculating the proportion of affected cases of each gene.

**Figure 4:**
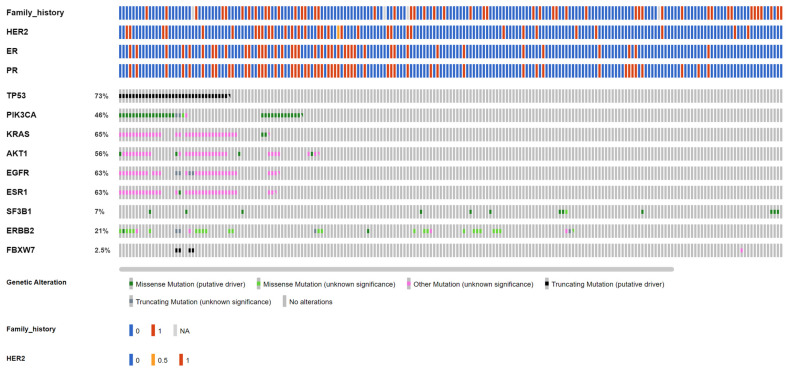
OncoPrinter of tumor tissue targeted sequencing from 246 early breast cancers. Two hundred and one patients reported at least one mutation, which was also the denominator when calculating the proportion of affected cases of each gene.

Among the 246 early-stage breast cancer patients (Group 1A from the VGH-TAYLOR study) assayed for both ctDNA and tumor tissue, the cfDNA assay detected 73 (29.6%, Figure 3) and the comprehensive assay detected 201 (81.7%, Figure 4) breast cancers with at least one variant (χ^2^ test, *p* = 0.001). A total of 121 and 1154 variants were detected from the liquid and tissue samples, respectively. 

In total, 67 (25.6%) cases tested positive for both the liquid and tissue assay, while the cfDNA and comprehensive assays detected an additional 10 (4%) and 138 (56%) cases, which were not identified by the other platform. Regarding allele frequency (AF), the median was 0.2% for *AKT1* (p.E17K); 0.08% for *ERBB3* (p.R103C, p.V104M); 1.21% for *KRAS* (p.G12A/D/V); 0.2% for *PIK3CA* (p.E545K, p.H1047R, p.M1043I, p.Q546K/R); 0.62% for *SF3B1* (p.K700E); and 0.11% for *TP53* (58 variant entities) from liquid biopsy. On the other hand, the AF for tumor tissue was 35.76% for *AKT1* (p.E17K, p.L52R, p.Y18fs); 6% for *EGFR* (p.V592I, p.A755fs, p.N466fs, p.N756fs); 48.36% for *ERBB2* (p.A763fs, p.D769Y, p.I655V, p.L841V, p.N758fs, p.V777L); 6.86% for *ESR1* (p.F62L, p.E380Q, p.M297I); 27% for *FBXW7* (p.A304fs, p.D550*, p.Q95K, p.R339fs, p.S294fs, p.T532fs, p.N679fs, p.E471G); 27.81% for *KRAS* (p.G12D/V); 24.5% for *PIK3CA* (p.C420_P421del, p.C420R, p.D1029H, p.D350N, p.D549N, p.E542K, p.E545K, p.E726K, p.E80K, p.G1049R, p.H1047L/R, p.H419Y, p.M1043I, p.N1044K, p.N345I/K, p.Q546K/P/R, p.Q721fs, p.Y1021H, p.Y432fs); 23.66% for *SF3B1* (p.K700E, p.R625H, p.W658C); and 49.22% for *TP53* (70 variant entities). Table 2 details the distributions of the variants among the six most common targeted genes. 

Table 3 details the alterations and clinical presentations of both the ctDNA and tumor sequencing (excluding synonymous mutations). In general, the comprehensive assay detected more alterations than the liquid biopsy as no mutation was identifiable through the cfDNA assay in *EGFR*, *ERBB2*, and *ESR1*. It deserves notice that three missense mutations in ERBB3, p.R103C, and p.V104M were only detected by liquid biopsy. *AKT1*, *EGFR*, *ESR1*, and *SF3B1* mutations were more pronounced in the HR+/HER2- subtype, while *PIK3CA* and *TP53* were mutated more evenly across all IHC subtypes. We also observed an increased incidence of family history of breast cancer among *AKT1* and *SF3BI* (tissue) as well as *PIK3CA* and *TP53* (both tissue and ctDNA). Also, more than three-fourths of *ERBB2*-mutant breast cancers were clinically HER2-negative. 

### 3.3. Early-Stage Breast Cancer Cohort: Actionable Mutations

The most prevalent mutant genes from the liquid biopsy and tissue samples, collectively, were *TP53* (68.3%, n = 168) and *KRAS* (53.5%, n = 131), both of which are well-known cancer driver genes. From breast cancer actionability, *PIK3CA* (ESCAT Tier IA) was reported in 39.4% (n = 97), *AKT1* mutation (ESCAT Tier IIB) in 45.9% (n = 113), and *ERBB2* mutation (ESCAT Tier IIB) in 17.1% (n = 42) of early-stage Taiwanese breast cancers (Table 3).

## 4. Discussion

Circulating tumor DNA is free DNA bound to proteins in plasma that originates from tumors, and tumor-derived DNA may only be a small minority of total cfDNA present in plasma. Next-generation sequencing has been developed for the detection of ctDNA in clinical trials, with potentialities of selecting therapies in metastatic settings, interrogating clonal evolution, and monitoring therapy in both metastatic and early settings [14]. Most studies on ctDNA in breast cancer have been conducted for an advanced/metastatic setting, while in this study we tried to answer the question of whether pre-operative ctDNA testing could serve as a non-invasive surrogate for variants which were to be identified from tumor tissue after surgery if a high concordance rate was observed.

Based on the results of 246 early-stage Taiwanese breast cancers, only one-quarter of patients tested positive for both the cfDNA and comprehensive assay from the same subject, indicating that assay-specific sensitivity inevitably resulted in a diagnostic discrepancy of targeted sequencing. In addition, the source of nucleic acid for the NGS experiments mattered especially for an early-stage disease setting. A plausible explanation came from the fact that less ctDNA spillage from tumors was expected for early breast cancer patients, which might compromise the detectability of liquid biopsy. 

In order to enhance the sensitivity of liquid biopsy, a technology called “cancer personalized profiling by deep sequencing” (CAPP-Seq) has been developed in the past decade, which can achieve high sensitivity with personalized profiling by deep sequencing [15,16,17]. For example, in non-small-cell lung cancer, CAPP-Seq showed that the amount of ctDNA was highly correlated with tumor volume as well as residual disease and was considered able to forecast the effect of treatment earlier than imaging examinations [18]. Although our study design was not sophisticated enough to adopt CAPP-Seq, the LOD parameters of the cfDNA assay in our study are listed as below; SNVs/short indels: LOD down to 0.1% AF could be achieved with a sensitivity of >80% and specificity of >98% and TP53 whole-target SNVs/indels: 0.5% AF (looking at all bases within amplicons); CNV targets: detection as low as 1.4-fold change can be achieved [19]. Consequently, LOD should not be an excuse for discordance between liquid biopsy and tumor tissue sequencing. 

The most prevalent mutations with both platforms combined were *TP53* (68.3%) and *KRAS* (53.5%), both well-known cancer driver genes. Although both are currently not targetable for breast cancer, *TP53* is a gene very commonly mutated in both clonal hematopoiesis and tumors and is rarely representative of the germline Li–Fraumeni syndrome; consequently, routine germline testing may not be necessary for most patients with somatic *TP53* mutations [20]. *KRAS* mutations are observed across a variety of cancer entities, while the recent advent of the KRAS (G12C) inhibitor render *KRAS*-mutant tumors druggable [21,22]. For contemporary breast cancer actionability, *PIK3CA* mutation is a biomarker for the FDA-approved PI3Kα inhibitor alpelisib, *AKT1* mutation is indicated for agents such as capivasertib (AZD5363) and ipatasertib, and *ERBB2* mutation for tyrosine kinase inhibitor neratinib [23,24,25,26,27]. Our study did ascertain the value of targeted sequencing for breast cancer, even at an earlier disease setting.

It is not a coincidence that the guideline update for biomarkers suggested that testing for *PIK3CA* mutations should use samples from tumor tissue or ctDNA in plasma to determine eligibility for treatment with alpelisib plus fulvestrant, a selective estrogen receptor degrader. If no mutation is found in ctDNA, testing in tumor tissue, if available, should be used as this will detect a small number of additional patients with *PIK3CA* mutations [28]. Although PI3K inhibitors are indicated for late-stage, hormone-receptor-positive, HER2-negative breast cancers and the liquid biopsy detection rate was assumed to be much higher for advanced disease, it was found that patients from the SOLAR-1 trial displayed low concordance in *PIK3CA* mutations between ctDNA and tumor tissue (56%), and FDA-approved labeling recommends reflex tissue testing when PIK3CA mutation is not detected from liquid biopsy [29]. Despite the detection rate of liquid biopsy (PCR or NGS) being much lower than that of tumor tissue (34% versus 60%), liquid biopsy was still suggested as the starting point for mutation testing as it may represent the most recent sample during tumor evolution [30].

Cancer treatment is rapidly evolving towards personalized medicine with targeted therapy corresponding to molecular alterations that lead to tumor growth. Therefore, proper assessment of dominant variants driving tumor evolution is warranted when deciding which patients are eligible for specific treatments. The way to obtain enough and the most recent tumor tissue for molecular profiling is quite challenging. Therefore, liquid biopsy through ctDNA has been proposed as an alternative to archived tumor slices. In this study, we expanded the use of liquid biopsy to early breast cancer, which can be used to detect molecularly metastatic or minimally residual disease pre- and post-operatively, indicating aggressiveness not readily detectable by contemporary pathological and clinical staging systems. The genetic variants identified with ctDNA are postulated to be associated with treatment response and reveal the earliest sign of recurrence. In the current study, the low concordance between tumor tissue and liquid biopsy did not surprise us too much given the early-stage disease setting. It deserves notice, however, that there were still a few cases with identified variants from ctDNA only, such as *ERBB3* mutations, and these patients should be followed up longitudinally to understand the meaning and prognosis of the ctDNA-positive/tumor-tissue-negative phenomenon. We also observed that AF was much lower for liquid biopsy than for the tissue-based counterpart.

There are some limitations to this study. First, interrogated genes were not identical between the comprehensive and cfDNA assays. Unlike targeted sequencing assays performed by Foundation Medicine (Cambridge, MA, USA) or Illumina (San Diego, CA, USA) which provide almost the same region of interest between tumor sequencing and liquid biopsy, the targeted genes for the Oncomine cfDNA Assay were far less than one-tenth of the comprehensive counterpart. Second, liquid biopsy was performed pre-operatively while tumor tissues were obtained from definitive surgery (Group 1A). Different sources of liquid and tissue biopsy might result in a time lag of about 2 to 4 weeks, and fluctuations in ctDNA during this time interval could not be completely ruled out. Third, the prospective nature of the VGH-TAYLOR study needed longer follow-up to determine the prognosis of targeted sequencing, especially for those cases with ctDNA-only alterations. 

## 5. Conclusions

Only one-quarter of breast cancers were concordant between tumor and liquid biopsy from the same subject. Early-stage breast cancer might shed less ctDNA from the tumor and compromise the detectability of liquid biopsy. Our study showed that tumor tissue should be the source of actionable mutation detection for early breast cancers.

## Figures and Tables

**Table 1:** Full cohort analysis of liquid biopsy samples from the VGH-TAYLOR study.

Gene	AKT1	CCND1	EGFR	ERBB2	ERBB3	ESR1	FGFR1	PIK3CA	SF3B1	TP53
Altered variants	8	11	7	16	11	38	7	107	11	445
Altered variants collapsed to subjects	8	11	7	15	10	36	7	102	11	416
Amino acid change (case number)	E17K (8)	CNV(11)	L861Q(7)	CNV(16)	R103C(4), T335I(2), V104M(5)	D538G(12), E380Q(4), Y537C(1), Y537N(9), Y537S(12)	CNV(7)	C420R(3), E453K(2), E542K(11), E545G(1), E545K(28), E726K(1), G12C(4), G12D(4), G12V(1), G13D(1), H1047L(12), H1047R(28), H1047Y(1), M1043I(2), Q546R(8)	K700E(11)	A138D(1), A138V(3), A189D(1), C124*(1), C141Y(2), C176S(1), C238Y(2), C242S(1), C242Y(1), C275F(1), C275Y(1), E180*(3), E258K(1), E258Q(7), E286G(1), E286K(1), E339*(1), E339K(1), F54L(1), G154S(1), G244D(2), G244S(3), G245D(8), G245S(6), G245V(1), G266E(2), G266R(4), H178L(1), H178fs(1), H179L(4), H179R(2), H193R(2), H193fs(2), H214R(1), H365fs(23), I195T(7), I195fs(6), K132*(1), K132N(5), K132R(4), K381T(2), L137M(1), L145R(1), L14P(1), L194P(1), L323V(1), L32M(1), L35fs(1), M237I(20), M246V(2), N131K(1), P151A(5), P151S(1), P151T(2), P152L(4), P177R(1), P190L(1), P222T(1), P250R(4), P278R(1), P300T(1), P301fs(4), P318T(1), P82fs(2), P85fs(7), Q104*(2), Q136H(1), Q136fs(2), Q192*(2), Q317*(1), Q331H(1), Q331fs(9), Q38H(1), Q38R(1), R156C(1), R158C(1), R158H(2), R175G(2), R175H(11), R175L(6), R181H(1), R181L(1), R181S(1), R196*(2), R213*(10), R213L(1), R213Q(6), R248Q(31), R248W(10), R249K(1), R249S(10), R273C(10), R273H(12), R273L(1), R273P(1), R280T(7), R282W(9), R283H(1), R306*(7), R333H(1), R333fs(1), R335fs(1), R379C(1), S149Y(1), S215R(2), S241C(1), S241F(7), S378fs(4), S6P(1), S90fs(8), S94*(1), S9G(1), T140_C14(2), V143M(4), V147fs(1), V157F(1), V157I(1), V172F(1), V173E(5), V173L(8), V173M(6), V197G(1), V216L(1), V216M(2), V272M(10), V274L(2), V31I(1), V97G(1), V97fs(2), Y103H(1), Y220C(13), Y220N(1), Y234C(2)
Functionality (case number)	Missense (8)	Amp(11)	Missense(7)	Amp(16)	Missense(11)	Missense(38)	Amp(7)	Missense(107)	Missense(11)	Missense(338), truncting(105), inframe(2)
Clinical presentations										
Group(1,2A,3-1,3-2)	1:0:2:5	0:2:0:9	0:0:0:7	3:6:5:2	7:2:0:2	0:0:2:36	0:0:0:7	25:26:16:40	6:3:0:2	196:100:17:132
ER(+/−)	8:0	11:0	NA	2:14	9:2	24:14	NA	71:27 *	9:2	237:157 **
PR(+/−)	8:0	3:8	NA	2:14	6:5	17:7	NA	49:49 *	9:2	182:212 **
HER2(+/−)	0:8	6:5	NA	16:0	1:10	3:35	NA	40:58 *	2:9	98:296 **

ER: estrogen receptor, PR: progesterone receptor, HER2: human epidermal growth factor receptor II, CNV: copy number variation, Amp: amplification. * missing values in 9 samples, ** missing values in 51 samples.

**Table 2:** Distributions of variants from common genes (n = 6) between cell-free DNA (cfDNA) and the tumor-tissue-based comprehensive assay.

Gene			Case Number		
	cfDNA	Tumor Tissue	Both Detected	Both Undetected	Total Affected
*AKT1*	1	113	1	133	113 (45.9%)
*ERBB2*	0	42	0	204	42 (17.1%)
*KRAS*	4	120	3	115	131 (53.5%)
*PIK3CA*	15	93	1	149	97 (39.4%)
*SF3B1*	3	14	1	230	16 (6.5%)
*TP53*	65	146	43	78	168 (68.3%)

**Table 3:** Distributions of alterations from liquid biopsy (ctDNA) and tissue samples from Group 1A patients of the VGH-TAYLOR study.

Gene	AKT1		CCND1		EGFR		ERBB2		ERBB3	
	ctDNA	Tissue	ctDNA	Tissue	ctDNA	Tissue	ctDNA	Tissue	ctDNA	Tissue
Altered variants	1	19	-	-	-	7	-	38	3	-
Amino acid change (case number)	E17K(1)	E17K(16), L52R(2), Y18fs(1)	-	-	-	A755fs(3), N466fs(2), N756fs(1), V592I(1)	-	A763fs(1), D769Y(1), I655V(31), L841V(1), N758fs(3), V777L(1)	R103C(2), V104M(1)	-
Functionality (case number)	Missense(1)	Missense(18), truncating(1)	-	-	-	Missense(1), truncating(6)	-	Missense(34), truncating(4)	Missense(3)	-
Clinical presentations										
ER(+/−)	1:0	17:2	-	-	-	6:1	-	31:7	2:1	-
PR(+/−)	1:0	17:2	-	-	-	6:1	-	28:10	2:1	-
HER2(+/−)	0:1	0:19	-	-	-	0:7	-	8:30	0:3	-
FH(case number)	0	8	-	-	-	1	-	5	1	-
**Gene**	**ESR1**		**FGFR1**		**PIK3CA**		**SF3B1**		**TP53**	
	**ctDNA**	**Tissue**	**ctDNA**	**Tissue**	**ctDNA**	**Tissue**	**ctDNA**	**Tissue**	**ctDNA**	**Tissue**
Altered variants	-	4	-	-	16	103	3	14	94	207
Amino acid change (case number)	-	E380Q(1), F62L(2), M297I(1)	-	-	E545K(3), H1047R(8), M1043I(1), Q546K(1), Q546R(3)	C420R(2), D1029H(1), D350N(1), D549N(1), E542K(6), E545K(18), E726K(5), E80K(1), G1049R(2), H1047L(8), H1047R(43), H419Y(1), M1043I(2), N1044K(1), N345I(1), N345K(4), Q546K(1), Q546P(1), Q546R(1), Q721fs(1), Y1021H(1), Y432fs(1)	K700E(3)	K700E(12), R625H(2), W658C(1)	C135W(1), C275Y(1), E285K(1), G244S(1), G245D(3), G245S(2), G279E(1), H179R(1), H193R(1), H214R(1), H365fs(3), I195T(1), K372E(1), L145M(1), L194R(1), M237I(4), M246V(1), N131K(1), P151A(1), P152L(2), P152fs(1), P222L(1), P222T(1), P250L(1), P278T(1), P300T(1), P4L(1), P85fs(1), Q331fs(9), R158H(1), R175C(1), R175H(2), R181H(1), R196*(3), R213*(2), R213Q(2), R248Q(2), R248W(5), R249S(2), R273C(2), R273H(2), R282W(1), R335S(1), R379C(1), S215fs(1), S378fs(1), S94*(1), V173L(1), V216M(1), V272M(1), V97G(1), W146C(1), W91L(1), Y220C(3), Y220S(1), Y234C(1)	A276fs(1), C135W(1), C176Y(1), C238S(1), C238Y(1), C242fs(1), C275F(1), C275Y(3), C275fs(3), D148fs(1), E271*(1), E285A(1), E285K(1), E286A(1), E286G(2), E336*(1), G245S(3), G266R(1), G266T(1), H168fs(1), H179R(2), H179Y(1), H193L(1), H214fs(1), I195T(2), L114fs(1), L194R(1), L348fs(1), M237I(1), M246I(1), M246R(1), M246V(1), N200fs(1), N239*(1), N268fs(1), P128fs(1), P278S(1), P278T(1), P295fs(1), P316fs(1), P390fs(1), P72R(106), Q331*(1), Q52fs(1), R110fs(1), R174W(1), R175H(5), R196*(3), R248G(1), R248Q(3), R248W(2), R249G(1), R249W(1), R273H(1), R333fs(1), R337C(1), S241F(1), S314fs(1), S94*(1), T125R(1), V218fs(1), V274D(1), V73fs(14), W91*(2), Y220C(3), Y234C(2), Y236C(1)
Functionality (case number)	-	Missense(4)	-	-	Missense(16)	Missense(101), truncating(2)	Missense(3)	Missense(14)	Missense(66), truncating(22), other(6)	Missense(162), truncating(45)
Clinical presentations										
ER(+/−)	-	4:0	-	-	11:5	86:17	3:0	14:0	72:22	154:53
PR(+/−)	-	4:0	-	-	8:8	76:27	2:1	13:1	65:29	141:66
HER2(+/−)	-	0:4	-	-	3:13	14:89	0:3	0:14	19:75	43:164
FH(case number)	-	0	-	-	5	21	1	7	25	41

ER: estrogen receptor, PR: progesterone receptor, HER2: human epidermal growth factor receptor II, FH: family history of breast cancer. “*” here indicated “Ter” (termination) for amino acid change.

## Data Availability

All data have been included in the main text, tables, and figures of the study. Individual targeted sequencing raw data such as FASTQ, BAM, and VCF files are restricted for access for protection of individual genetic information. Further requests for these data can be made to the corresponding author, with additional review from the Internal Review Board.
